# Short-Term Compassion Training Increases Prosocial Behavior in a Newly Developed Prosocial Game

**DOI:** 10.1371/journal.pone.0017798

**Published:** 2011-03-09

**Authors:** Susanne Leiberg, Olga Klimecki, Tania Singer

**Affiliations:** 1 Laboratory for Social and Neural Systems Research, Department of Economics, University of Zurich, Zurich, Switzerland; 2 Department of Social Neuroscience, Max Planck Institute for Human Cognitive and Brain Sciences, Leipzig, Germany; University of Granada, Spain

## Abstract

Compassion has been suggested to be a strong motivator for prosocial behavior. While research has demonstrated that compassion training has positive effects on mood and health, we do not know whether it also leads to increases in prosocial behavior. We addressed this question in two experiments. In Experiment 1, we introduce a new prosocial game, the Zurich Prosocial Game (ZPG), which allows for repeated, ecologically valid assessment of prosocial behavior and is sensitive to the influence of reciprocity, helping cost, and distress cues on helping behavior. Experiment 2 shows that helping behavior in the ZPG increased in participants who had received short-term compassion training, but not in participants who had received short-term memory training. Interindividual differences in practice duration were specifically related to changes in the amount of helping under no-reciprocity conditions. Our results provide first evidence for the positive impact of short-term compassion training on prosocial behavior towards strangers in a training-unrelated task.

## Introduction

Prosocial behavior is a pervasive aspect of human life: We cooperate with others and help them when they are in need. However, diametrically opposed to these behaviors are everyday experiences of people taking advantage of others. The present study is concerned with the question whether compassion training can increase prosocial behavior. Compassion has been defined as the emotion one experiences when one feels concern for another's suffering and desires to enhance that person's welfare ([1], see [2] and [3] for more detailed definitions). In the present paper, we use the term “compassion” to describe an emotional as well as a motivational state, characterized by feelings of warmth, love, and concern for the other as well as the desire to help and promote the other's welfare. The term “empathic concern” has been used in a very similar way in developmental and social psychology [4], [5]. For example, Batson [6] maintains that empathic concern “is an other-oriented emotional response elicited by and congruent with the perceived welfare of someone in need involving feelings *for* the other such as sympathy, compassion, tenderness, and the like.” However, while empathic concern mainly denotes a situation-specific, rather short-living emotion, compassion can also be thought of as an attitude [7]. Empirical evidence suggests that empathic concern is a perpetuator of prosocial behavior [8], [9]. For example, it has been demonstrated [10] that momentarily inducing feelings of empathic concern for a person in need by having participants focus on the person's feelings increases their prosocial behavior towards that person. More specifically, participants who were instructed to feel empathic concern for a person receiving painful electric shocks were willing to receive more shocks themselves to alleviate the other person's suffering than participants who had been encouraged to remain detached. The effects of this situation-specific induction of empathic concern, however, are probably rather short-lived and might not extend over the particular experimental session. Furthermore, it is not clear whether the induction of empathic concern for a specific person leads to increases of prosocial behavior only for that specific person [e.g. 6] or whether it generalizes to different persons as well [11], [12]. While the experimental induction of empathic concern through explicit perspective-taking instructions or listening to songs with prosocial lyrics [11] might temporarily prime people to experience empathy when seeing the distress of others, training of compassion aims at permanently changing people's motivation and their feelings towards other people. It strives to develop a more friendly, benevolent, connected and positive attitude towards others. In the long run, compassion training-induced changes at the trait level – but not at the state level - might even take effect on the opiate- and oxytocin-based affiliative system [7], [13].

We hypothesize that, contrary to a short-term instruction-based induction of empathic concern towards a specific person, compassion training will elicit a longer-lasting enhancement of general compassionate motivation, which in turn may lead to an increase in the general tendency to act prosocially, independent of person and situation.

Even though there is a long history of behavioral plasticity research pertaining to the training of cognitive [14], perceptual [15], motor [16] as well as affective skills [17], [18], no study to our knowledge has investigated behavioral changes resulting from compassion training. Thus, in one study, for example, empathy for a personal offender was trained over eight 1-hour sessions and an increase in reported empathy and forgiveness but not prosocial behavior was measured [19]. Similarly, the few existing studies on compassion training have examined the effects of compassion training on mood and health but not prosocial behavior [20]–[23]. In a pilot study, Gilbert and Procter [22] administered compassionate mind training [CMT; 24], which aims at reducing self-criticism by focusing on compassionate images and emotions, to a sample of psychiatric patients with severe long-term difficulties. They reported reductions in depression and anxiety as well as increases in self-soothing abilities and feelings of warmth for oneself. Other studies investigating the effects of compassion training have used meditation-based techniques that involve the development of warm, positive feelings towards a variety of people and ultimately towards all human beings: Six to seven weeks of meditation-based compassion training result in increases in positive mood and life satisfaction [21] as well as a reduction of interleukin-6 release in response to a psychosocial stressor [23]. The more time participants had actually spent training, the stronger the reduction in interleukin-6 release, suggesting a dose-dependent effect of compassion training. Hutcherson et al. [25] report that a very brief (7-min) compassion meditation exercise results in a more positive attitude towards the target of the exercise. Taken together, these studies provide promising support for the health- and positivity-promoting effects of compassion training. However, so far, no study has investigated whether prosocial behavior can actually be increased through compassion training and whether the practice of compassion promotes a generalized tendency for prosocial behavior. Thus, the aim of the present study was to investigate the effect of short-term compassion training on prosocial behavior.

In behavioral economics, prosocial behavior is usually studied in the context of well-controlled monetary exchange games [26] and mostly explained in terms of social preferences or norms, such as fairness and reciprocity [27], whose evolution has also been linked to reputation concerns [28], [29]. It could also be shown that observation of prosocial behavior in a public goods game with multiple rounds increases the likelihood of later prosocial behavior of the observer towards another person in the following rounds [30]. However, the influence of compassion or empathy and their training on prosocial behavior has so far never been discussed or studied in the field of economics. In the context of game theoretical paradigms, the dictator game is most commonly used for assessing altruistic acts towards others [31]–[34]. In this game, participants are endowed with a sum of money that they can split between themselves and another participant who has no money. Giving in the dictator game is likely driven by fairness norms and not by kindness [35]. While several motives have been discussed as underlying prosocial behavior, only recently a differentiation between norm-based and compassion-based prosocial behavior has been suggested [2]. While the former is particularly encountered in “cold”, reasoning-driven exchange situation, the latter is often present in “hot”, emotion-provoking situations. Compassion training might take its effects on the latter but not the former. Since many of our everyday interactions are not purely rational, but involve emotions, an adequate paradigm that assesses prosocial behavior in an engaging, ecological setting and that is sensitive to affective interventions needs to be developed. This paradigm would moreover allow for future investigation of the proposed differentiation between norm-based and compassion-based prosocial behavior.

In social psychology, prosocial behavior is mostly assessed in emotion-provoking one-shot helping situations of high ecological validity, such as dropping pens, soliciting donations for charities, or soliciting help with filling out or scoring questionnaires [6], [36]–[38]. However, these paradigms as well as the above-mentioned economic paradigms do not allow for the repeated assessment of prosocial behavior within the same person, which is required in intervention studies with multiple measurement time points such as the present study. We therefore developed a new prosocial task – the Zurich Prosocial Game (ZPG) – that allows for the repeated assessment of prosocial behavior within the same person while still being ecologically valid, and thus being suitable to investigate changes in prosocial behavior due to compassion training.

In addition the new game was developed to simultaneously assess the influence of reciprocity, the cost associated with helping, and distress cues on prosocial behavior. It has been shown that people help more often if they have been helped before [39], [40], if the costs of helping are low [41] and if they are confronted with signs of distress [42], [43]. These factors are of interest as evolutionary biologists and anthropologists demonstrated that they are selected for in evolution and provide a biological basis for altruism. Reciprocal altruism evolved as a costly altruistic act which might be repayed at a later time [44], costly helping is mostly directed towards kin as suggested by the model of inclusive fitness [45] and distress cues, such as crying, evolved to signal the need for help and to sustain close personal bonds [46]. The possibility to distinguish between these helping-related factors within one task allows the investigation of differential effects of context, intervention or personality on different helping conditions in future research. Here, the aim was first to test the effect of a short-term compassion training on prosocial behavior in the ZPG.

To validate the newly developed task and to test the effects of compassion training on prosocial behavior, we performed two independent experiments. The first experiment was conducted to validate the newly developed prosocial task, the so-called Zurich Prosocial Game (ZPG) and to test its sensitivity to the influence of reciprocity norms, helping costs and distress cues on helping. We hypothesized that people would help more a) if they had been helped before, b) if the cost of helping was low, and c) if they were confronted with distress cues. The second experiment was conducted to investigate the influences of short-term compassion training on prosocial behavior towards strangers as measured by the ZPG – a game that is completely unrelated to the training context. We hypothesized that short-term compassion training leads to stronger increases in helping than a short-term memory training, the latter received by a control group. Furthermore, time spent practicing the compassion-enhancing technique should be positively correlated with this increase in helping. Based on the suggested distinction between compassion-based and norm-based prosocial behavior, and on the assumption that the compassion training has effects on the former this correlation could possibly only arise for non-reciprocity trials.

## Results

### Experiment 1

**Figure 1:**
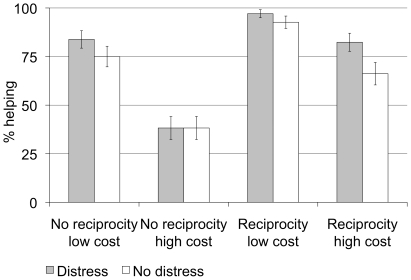
Percent helping in the different conditions of the ZPG. Error bars denote standard errors of mean.

To investigate the effects of reciprocity, cost, and distress on the occurrence of prosocial behavior, we computed a 2 (reciprocity: no reciprocity, reciprocity) x 2 (cost: low, high) x 2 (distress: no distress cues, distress cues) within-subjects repeated-measures analysis of variance (ANOVA). This analysis revealed main effects of reciprocity, cost, and distress (see Table 1). As hypothesized, participants helped significantly more in reciprocity trials, in trials with a low cost of helping, and in trials in which the co-player's virtual character expressed distress (see Materials and Methods for a detailed description of the ZPG). Thus, the ZPG indeed seems to be sensitive to the three operationalizations of the influencing factors, which suggests that reciprocity, distress cues, and low cost are associated with increased helping behavior (see Figure 1).

**Table 1:** ANOVA for the effects of reciprocity, cost and distress cues on prosocial behavior in experiment 1 and 2.

Source	df	F	partial *η^2^*	*p*
Experiment 1				
Reciprocity	67	73.22	.52	<.001
Cost	67	73.78	.52	<.001
Distress	67	7.02	.10	.01
Reciprocity x Cost	67	13.21	.17	.001
Reciprocity x Cost x Distress	67	4.10	.06	<.05
Experiment 2				
Reciprocity	68	51.55	.43	<.001
Cost	68	66.04	.49	<.001
Reciprocity x Cost	68	7.96	.11	<.001
Reciprocity x Distress	68	6.11	.08	<.05

All main effects and interactions significant on a p<.05 level are reported.

The main effects of reciprocity and cost were qualified by a significant interaction between the two factors (see Table 1). Increasing the cost of helping resulted in a larger decrease in prosocial behavior in the no-reciprocity compared to the reciprocity trials, suggesting that norms such as reciprocity can absorb the decline in prosocial behavior when helping is costly. Furthermore, there was a three-way interaction between the three factors (see Table 1). Separate follow-up ANOVAs for distress and no-distress trials revealed that the reciprocity x cost interaction was only significant in the distress trials, *F*(1,67) = 17.73, *p*<.001, partial *η^2^* = .21.

To confirm that interindividual differences in helping behavior as measured using the ZPG are not brought about by differences in allocation of attention, we calculated the correlation between the total amount of helping and the percentage of stars picked up, that randomly appeared during the game. On average, participants picked up the star in 42.6% of the trials in which a star appeared. A star appeared in two to six (of nine) trials. The non-significant correlation, *ρ*(66) = −.07, *p*>.05, between total amount of helping and percentage of stars picked up indicates that differences in attention allocation most likely do not account for interindividual differences in prosocial behavior.

Furthermore, to control for potential effects of individual differences in risk preferences on helping in the high cost trials, the risk questionnaire and the lottery index were correlated with helping in high cost trials (see Materials and Methods for a description of the risk perception control measures). As both correlations were non-significant, we can preclude that interindividual differences in risk preferences, *ρ*(66) = .10, *p*>.05 (risk questionnaire) and *ρ*(66) = .02, *p*>.05 (lottery index), accounted for the difference in prosocial behavior between low and high cost trials.

Furthermore, participants had to judge their engagement in the game (see Materials and Methods). The analyses of these subjective engagement scores revealed that on average participants indicated that they were very engaged in the game (range: 2–5; mean: 4.15, SD: 0.74); a result which matches the observation of the experimenter who reported that the subjects were all very immersed in the ZPG.

To assess the convergent validity of the ZPG, participants played the dictator game [31] (see Materials and Methods). Based on our reasoning about norm-based and compassion-based prosocial behavior in the introduction, we did not expect an exceedingly high correlation between the ZPG and the dictator game, but still, as both tasks assess variants of prosocial behavior, a sufficiently high correlation to maintain that the ZPG indeed measures prosocial behavior. In the dictator game, participants gave 36.29% of their endowment on average. Most of the participants (40.3%) gave half of their endowment and 10.4% gave nothing. As expected, giving behavior in the dictator game correlated with helping behavior in the ZPG, *ρ*(65) = .35, *p* = .004, substantiating the validity of our game as a measure of prosocial behavior.

To assess the divergent validity of the ZPG, we used a memory task (see Materials and Methods). Participants remembered 18.24 words (standard deviation [sd]: 5.86) on average in the memory task. The number of remembered words did not correlate significantly with helping in the ZPG, *ρ*(64) = .06, *p*>.05, demonstrating divergent validity of the ZPG.

### Experiment 2

In the following, we will first present the pre-training data from the newly developed ZPG to ascertain whether the results found in Experiment 1 are robust. We will then report data on the effectiveness of the compassion training workshop and on the effects of compassion training on prosocial behavior in the ZPG. The effects of compassion training were tested one-sided as we had clear hypotheses about the direction of effects (see Introduction).

#### Robustness of ZPG

To investigate the robustness of the result pattern in the ZPG, we computed a 2 (reciprocity: no reciprocity, reciprocity) x 2 (cost: low, high) x 2 (distress: no distress cues, distress cues) within-subjects repeated-measures ANOVA for the total sample of Experiment 2 (compassion group and memory group). The analysis again revealed main effects of reciprocity, and cost (see Table 1). Participants helped significantly more in reciprocity trials and in trials with a low cost of helping. As in Experiment 1, these main effects were qualified by a significant interaction between the two factors, again suggesting that norms such as reciprocity can absorb the decline in prosocial behavior when helping is costly. In contrast to Experiment 1, however, no main effect of distress was observed, *F*(1,68) = 1.89, *p*>.05, partial *η^2^* = .03. The analysis did however yield a reciprocity x distress interaction: Distress cues increased helping in the no-reciprocity but not in the reciprocity trials, which might indicate that no-reciprocity trials are more sensitive to other influencing factors (see Table 1).

We again did not observed a significant correlation between total amount of helping and percent of stars picked up, *ρ*(67) = −.12, *p*>.05, indicating that differences in attention allocation most likely do not account for interindividual differences in helping behavior.

As in Experiment 1 the correlations between helping in the high cost trials with both the risk questionnaire, *ρ*(57) = .02, *p*>.05, and the lottery index, *ρ*(57) = .05, *p*>.05, were non-significant, precluding that interindividual differences in risk preferences accounted for the difference in prosocial behavior between low and high cost trials.

Participants in experiment 2 also reported to be very engaged in the game (range: 1–5; mean: 4.04, SD  = 1.07). There was no differences between participants in the compassion and memory training group in the engagement with the game, t(52) = 1.78, p>.05, suggesting that potential differences between the groups cannot be accounted for by differences in motivation and degree of being emerged into the game.

In the dictator game, on average, participants gave 33.8% of their endowment. Most of the participants (39.4%) gave half of their endowment and 13.6% gave nothing. More importantly, giving behavior as measured with the dictator game again correlated with helping behavior as measured with the ZPG, *ρ*(67) = .45, *p*<.001.

Participants remembered on average 20.53 words (sd: 7.04) in the memory task. And as in Experiment 1, the number of remembered words did not correlate with helping in the ZPG, *ρ*(66) = −.04, *p*>.05, giving repeated evidence for divergent validity of the ZPG.

#### Effectiveness of compassion training

Repeated-measures ANOVAs with time (pre-training, post-training) as a within-subjects factor and training (compassion, memory) as a between-subjects factor were calculated to determine the effectiveness of the compassion training in enhancing self-reported positive (assessed with the Positive and Negative Affect Scale [PANAS; 47]) and compassionate (assessed with the Compassionate Love Scale [CLS; 48]) feelings and reducing negative feelings (also assessed with the PANAS [47]). A significant main effect of time on positive mood, *F*(1,54) = 23.47, *p*<.001, partial *η^2^* = .30, was revealed, indicating that compassion training as well as memory training increased positive mood. A significant main effect of time, *F*(1,54) = 5.84, *p* = .02, partial *η^2^* = .10, was revealed for compassionate feelings that was qualified by a marginally significant interaction between time and training, *F*(1,54) = 3.61, *p* = .06, partial *η^2^* = .06. Post-hoc t-tests indicated that only the compassion-training group experienced a significant increase in compassionate feelings, *t*(23) = 2.66, *p* = .01. For negative mood, a significant time x training interaction was revealed, *F*(1,68) = 6.11, *p* = .016, partial *η^2^* = .08. While negative mood decreased in the compassion-training group, *t*(23) = −1.94, *p* = .03, one-sided, it marginally significantly increased in the memory-training group, *t*(23) = 2.02, *p* = .05.

#### Effect of compassion training on prosocial behavior

To test whether a brief compassion training had an effect on prosocial behavior in the ZPG, we conducted two analyses: First, we performed a 2 (time: pre-training, post-training) x 2 (reciprocity: no reciprocity, reciprocity) x 2 (cost: low, high) x 2 (distress: no distress cues, distress) repeated-measures ANOVA with training (compassion, memory) as a between-subjects factor. Second, we tested for increases in helping as a function of interindividual differences in hours of reported training. To this end, we calculated the correlation between participants' self-reported time spent praticing outside of the training and the change in helping from pre- to post-training (self-report data could only be obtained from a subset of the samples: *n*_compassion_ = 19, *n*_memory_ = 22).

**Figure 2:**
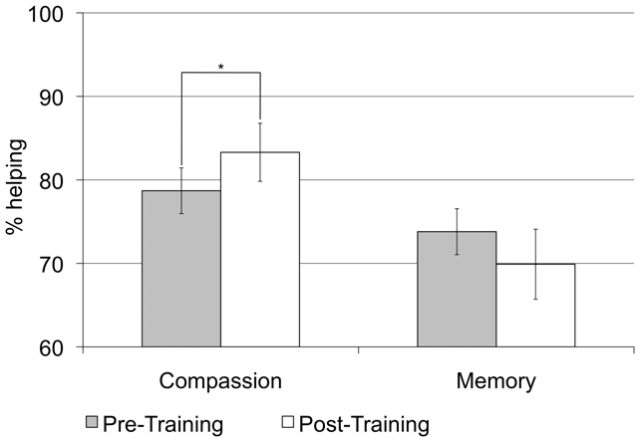
Effects of training on overall helping in the ZPG for the compassion-training and memory-training group. Error bars denote standard errors of mean. * p<.05, one-sided.

In the first analysis, we observed a significant time x training interaction, *F*(1,57) = 4.09, *p* = .05, partial *η^2^* = .07. While there was no reliable change in helping from pre- to post-training for the memory training group, *t*(31) = −1.20, *p* = .24, compassion training significantly increased helping, *t*(26) = 1.85, *p* = .04, one-sided (see Figure 2). Additionally, a time x cost interaction was observed, *F*(1,57) = 6.76, *p* = .01, partial *η^2^* = .11. These interactions were qualified by a significant three-way interaction between time x cost x training, *F*(1,57) = 4.55, *p* = .04, partial *η^2^* = .07. Follow-up independent t-tests indicated that, at pre-training, helping in the low-cost, *t*(57) = 1.21, *p* = .23, and high-cost trials, *t*(57) = .55, *p* = .58, did not differ between the compassion and the memory group whereas, at post-training, the groups differed significantly in helping in both the low-, *t*(57) = 3.07, *p* = .003, and the high-cost trials, *t*(57) = 2.27, *p* = .03.

The second analysis did not reveal the hypothesized correlation between interindividual differences in reported hours of compassion training and total helping, *ρ*(17) = .27, *p* = .13, one-sided. To test our expectation that interindividual differences in reported training hours are differentially related to the different trial types, affecting more non-reciprocity than reciprocity trials, we calculated the correlation between training hours and helping in the different trial types and found a significant correlation between interindividual differences in reported hours of compassion training and helping in no-reciprocity trials, *ρ*(17) = .39, *p* = .05, one-sided. Correlations with the other trial types as well as all correlations in the memory group were non-significant at *p*<.05, one-sided.

To investigate whether compassion training could also increase giving in the dictator game, we computed a repeated-measures ANOVA with time (pre-training, post-training) as a within-subjects factor and training (compassion, memory) as a between-subjects factor. Interestingly, neither a significant main effect of time, *F*(1,56) = .02, *p* = .89, partial *η^2^*<.001, nor an interaction between time and training *F*(1,56) = 1.63, *p* = .21, partial *η^2^*<.001, was observed. Participants in neither group gave more money to the other person after training compared to before training.

## Discussion

The present study introduces a newly developed prosocial game – the Zurich Prosocial Game (ZPG) – and provides first evidence for the effectiveness of short-term compassion training in enhancing prosocial behavior in this new training-unrelated game towards strangers.

The ZPG was developed as previous prosocial tasks from behavioral economics or social psychology are either not particularly ecologically valid or do not allow for the repeated assessment of prosocial behavior which is required in intervention studies with multiple measurement time points. The ZPG extends the prosocial tasks from behavioral economics and social psychology in several aspects. First, the influence of reciprocity, cost, and distress on prosocial behavior has been studied separately before, but the ZPG now has the advantage to allow their simultaneous assessment in the same setting. This is particularly useful when studying the differential influence of experimental manipulations on these factors. Second, while many prosocial tasks are only applicable one time, the ZPG can be played multiple rounds and on different time points with the same subjects thus allowing for more stable estimates of prosocial behavior, the usage in neuroscientific settings where many trial repetitions are needed, and, the assessment of changes in prosocial behavior over time through interventions. Third, the ZPG is more ecologically valid than, for example, monetary exchange games, as it minimizes the influence of strategic considerations, minimizes effects of task-affordances due to explicit instructions and maximizes the influence of emotion-driven, fast decisions, since participants are immersed in the game itself whose explicit goal is to achieve a treasure in short time rather than act prosocially. In the ZPG participants help others by spending ressources (key, time) they might need later on. This type of prosocial behavior that involves uncertainty for oneself can be encountered often in daily life, for example when we run for an important appointment and see someone fall from his bike. Do we stop to help this person without knowing the outcome and how much time it will take or do we refrain from helping and make sure that we reach our appointment on time? And fourth, as the ZPG is very engaging and easy to use, it is also very well suited to study prosocial behavior in children.

The present results confirm that the ZPG is sensitive to influences of reciprocity, cost, and distress on prosocial behavior: As predicted, participants of two independent experiments helped more when having been helped before and when costs are low. Interestingly, the drop in prosocial behavior with increasing costs was less pronounced when participants had been helped before suggesting that norms of reciprocity override cost considerations. While in Experiment 1 participants helped more when confronted with distress cues, in Experiment 2, distress cues increased helping only in the no-reciprocity trials but not in the reciprocity trials. This may again suggest that reciprocity norms are so pervasive that they overrule the effect of any other influencing factor, whereas prosocial behavior without reciprocation is more affected by other factors. In both samples, the convergent validity with a well-established economic prosocial task, the dictator game, was confirmed. This supports our claim that the new game does indeed assess prosocial behavior. The correlation between the two tasks, however, is modest, suggesting that the two measures tap into different aspects of prosocial behavior. Furthermore, divergent validity was established with a memory task.

In Experiment 2, we were able to demonstrate that compassion training but not memory training significantly increased helping in the ZPG. Previous studies have demonstrated that a momentary instruction-based induction of empathic concern for a specific person increases prosocial behavior towards that person immediately after induction [8]. Here we show for the first time that compassion training had longer-lasting effects on prosocial behavior as the post-test was completed two to five days after training. Furthermore, short-term training resulted in transfer to behavior in a novel task that was completely unrelated to the previous affective training. Finally, compassion training increased prosocial behavior towards people who were not specifically targeted during training but complete strangers to the participants.

The present results support the notion that similar to situation-specific induction of empathic concern for a specific person in need [8], [49], the training of compassionate motivation leads to increases in prosocial behavior. In comparison to experimental inductions of empathic concern, however, compassion training has the potential to lead to longer-lasting changes in people's attitude and behavior towards other people that transcend the specific situation in which compassionate feelings were evoked and transfer to a much broader range of people and situations.

Self-benefiting effects of compassion training such as increases in positive mood, life satisfaction, decreased depressive symptoms [21], and less reactivity to psychosocial stress [23] have been reported before. The present study adds to these findings by showing that even a short-term compassion training may not only have benefits for the practitioner's health and subjective well-being but also for other people and society in general as it increases the propensity to act prosocially even towards people one has never met. Notably, the prosocial behavior observed here was not directed towards a target of the compassion training but to random strangers and was assessed at least two days after the training, which lends further credibility to the societal impact that the implementation of compassion training in schools, organizations, and clinical settings might have (for compassion training in psychotherapy, see [7]).

Another interesting finding of the present study was that helping in no-reciprocity trials, but not helping in reciprocity trials, was related to interindividual differences in reported training hours in the compassion group. This might provide tentative evidence for a differentiation between compassion-based and norm-based prosocial behavior as has been suggested before [2]. Accordingly, helping after having been helped may rely on a felt obligation to reciprocate cooperation. In contrast, helping without the possibility for reciprocity may be motivated more by feelings of compassion than by “cold” norms. The pattern of correlations found here suggests that compassion training might have differential effects on both types of underlying motivation. This is further supported by our finding that giving in the dictator game did not change from pre- to post-training in either group and that the modal giving at pre-training was 50%. Moreover, the correlation between helping in the ZPG and giving in the dictator was modest suggesting that the two measures tap into different aspects of prosocial behavior. Giving in the dictator game has previously been shown to depend more on fairness norms than on kindness [35]. These findings suggest a distinction between compassion-based and norm-based prosocial behavior with compassion training possibly exerting a stronger effect on the former than on the latter. As the current study was not designed to test the hypothesis of a distinction between compassion-based and norm-based prosocial behavior, future investigations are needed. For example, using priming of a reciprocity-norm or compassion could be used to show a differential effect of these concepts on different helping settings. The simultaneous assessment of reciprocity and non-reciprocity driven prosocial behavior in the ZPG makes this game ideal for this aim. Similarly, investigations with longer training will be of great interest. As the results suggest, the novel ZPG might be a better measure for assessing training-induced changes in prosocial behavior, specifically compassion-based prosocial behavior, than standard economic games or psychological measures as we were able to show that it is more sensitive to change than, for example, the dictator game. The higher sensitivity to changes in compassion-based prosocial behavior might result from the high emotional engagement participants experience when playing the ZPG.

Importantly, as the game is framed as a treasure hunt with monetary gains, demand effects induced by the content of the training should be less strong than in other prosocial tasks. While in economic games the sharing purpose is made explicit, here the instruction focuses on the rules of the game and emphasize that the goal is to reach the treasure in a limited time while having to overcome certain obstacles. Furthermore, the game is very engaging (on average participants rate their involvement in the game with 4 on a 1-to-5-scale and report later that they find the game very enjoyable) and fast, thus making strategic considerations difficult. Compassion training not only increased prosocial behavior but also led to increases in reported compassionate feelings and positive affect and a decrease in negative affect. Interestingly, the memory-training group also evinced an increase in positive mood, suggesting that increases in positive mood are not sufficient for explaining enhanced prosocial behavior. We maintain that compassion training enhanced prosocial behavior through initial changes to participants' way of feeling and thinking about other people to a more positive, benevolent and friendly attitude. This is in line with participant's qualitative post-study reports of being more sensitive to others, feeling more connected, secure and open and having “a bigger and more open heart.” The present study provides first evidence for compassion training but not memory training causing increases in prosocial behavior. Future studies should elucidate, which aspects of the training led to the observed effect. Apart from the suggested change in other-related attitudes, increased relaxation or feeling of oneness (perceived self-other overlap; [50]) could be additional mechanisms through which compassion training increases prosocial behavior.

In sum, the present study provides first evidence for the effectiveness of a short compassion training in increasing prosocial behavior in a newly developed computer task, the Zurich Prosocial Game. Using this novel training-unrelated computer task, we found that compassion training that aimed at fostering a friendly, benevolent attitude towards others produced a significant increase in prosocial behavior two to five days after training towards strangers. Interestingly, practicing compassion strategies seems to influence compassion-based prosocial behavior more strongly than norm-based prosocial behavior. The effectiveness of the compassion training was further supported by an increase in positive mood and compassionate feelings and a decrease in negative mood. Future research with longer training and bigger sample sizes needs to ascertain how long lasting these effects are and who is benefitting from compassion training. Clinical research for example suggests that some people find compassion-focused imagery distressing [51], [52] and thus do not benefit from it. Furthermore it needs to be investigated whether long-term compassion training leads to stronger increases in specific types of prosocial behavior and whether this effect can also be observed in everyday life behavior. As the interpersonal effects were directed towards total strangers and transferred to situations outside the training context, compassion training could have great societal impact when implemented in institutions of daily life.

## Materials and Methods

### Participants

In Experiment 1, that aimed to validate the newly developed ZPG, we investigated 68 healthy female volunteers (aged 18–35 years; mean: 25.18; years of education after the 16^th^ birthday: 2–15 years; mean: 6.54). In Experiment 2, that aimed to assess the effect of a short-term compassion training workshop on prosocial behavior as measured using the newly developed ZPG, we investigated 69 healthy female volunteers (age: 18–34 years; mean: 23.69). Only female participants were included in Experiments 1 and 2 because of better performance in emotional tasks [53] and higher self-reported empathy in women [54]. All participants came from the University of Zurich and the surrounding communitya and were recruited through local advertisement and internet postings. The advertisements for Experiment 2 asked for people interested in mental training but never mentioned the word compassion. All participants completed the Toronto Alexithymia Scale [TAS; 55], the Beck's Depression Inventory [BDI; 56] and sociodemographic questions online. Only when they met the following inclusion criteria they were contacted via telephone: aged 18–35 years, TAS <60, BDI <18, right hander and no contraindication for fMRI. Importantly, possible participants of Experiment 2 were additionally not allowed to have prior experience with mental compassion training or the method of loci. On the phone, participants were given information about the timing but, importantly, in case of Experiment 2, not about the specific content of the study and underwent a structured psychological interview (screening questions for axis-I disorders and psychotic disorders of the Structured Clinical Interview for DSM Disorders [SCID; german version: 57]). Woman with current psychiatric illnesses were excluded from the study. For Experiment 2, allocation to the compassion-training and memory-training (control) group depended on slot availability and time of the participants. 35 participants entered the compassion-training group and 34 participants entered the memory-training group. 28 participants from the compassion group and 32 participants from the memory group completed the study. One participant of the compassion group was eliminated from the analysis as data on the ZPG was missing. The majority of the dropout in the compassion group (5/7) occurred before the training. Furthermore, the seven participants that dropped out of the study did not differ in age, *t*(32) = .57, *p*>.05, years of education, *t*(32) = 1.75, *p*>.05, empathic concern, *t*(32) = −.81, *p*>.05, alexithymic symptoms, *t*(32) = 1.70, *p*>.05, depressive symptoms, *t*(32) = −.75, *p*>.05, prosocialness, *t*(32) = −.79, *p*>.05, compassionate feelings, *t*(32) = −.99, *p*>.05, and general positive, *t*(32) = 1.42, *p*>.05, and negative affect, *t*(32) = −.06, *p*>.05, from the participants that finished the study, thus excluding selective dropout in the compassion group.

The compassion group and the memory group did not differ in age, *t*(57) = 1.98, *p*>.05, years of education, *t*(57) = .64, *p*>.05, prosocialness, *t*(54) = 1.91, *p*>.05, empathic concern, *t*(54) = .1, *p*>.05, alexithymic symptoms, *t*(57) = .89, *p*>.05, or depressive symptoms, *t*(57) = .87, *p*>.05. There was also no difference in the distribution of type of education between the samples (*χ^2^* = 3.06, *p*>.05). See Table 2 for sample characteristics. The study was approved by the Research Ethics Committee of Zurich (“Kantonale Ethikkommission des Kantons Zürich – Spezialisierte Unterkommission Psychiatrie, Neurologie, Neurochirurgie”; E-25/2008) and was performed according to the Declaration of Helsinki. All participants gave written informed consent after having received a full description of the study.

**Table 2:** Sample characteristics.

	Validation sample (N = 68)	Compassion training sample (N = 27)	Memory training sample (N = 32)
Age	25.18 (4.08)	24.74 (4.22)	22.66 (3.86)
Highest completed education	Apprenticeship: 5 (7.5%) High school: 34 (50.8%) University: 28 (41.8%)	Apprenticeship: 3 (11.1%) High school: 18 (66.7%) University: 6 (22.2%)	Secondary school: 1 (3.1%) Apprenticeship: 1 (6.3%) High school: 24 (75%) University: 4 (12.6%) PhD: 1 (3.1%)
Education (years after 16^th^ birthday)	6.54 (2.87)	5.48 (2.44)	5.06 (2.54)
Prosocialness^1^	64.03	60.75	64.50
Empathic concern^2^	27.64	27.08	27.19
Alexithymia^3^	41.24	39.41	41.16
Depression^4^	6.13	6.04	4.53

1 Prosocialness Scale [61] (range: 16–80).

2 Empathic Concern Subscale Interpersonal Reactivity Index [4] (IRI; range: 7–35).

3 Toronto Alexithymia Scale [53] (TAS;>60 clinically relevant).

4 Beck's Depression Inventory [54] (BDI;>18 clinically relevant).

### Measures

#### Zurich Prosocial Game

**Figure 3:**
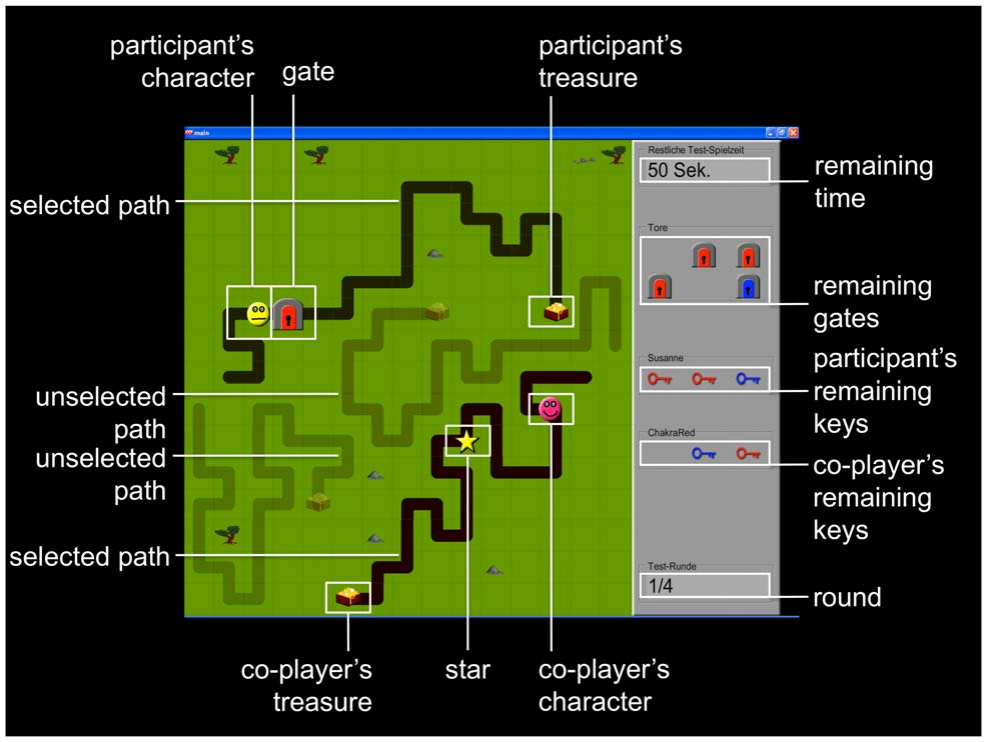
Labeled screenshot of the ZPG. Participants move their virtual character forward by clicking with the mouse on the field in front of it. Usage of keys in order to open the blocking gates occurs by mouse click on the key matching the gate's color. Collection of stars also occurs by clicking on them with the mouse.

A novel game, the Zurich Prosocial Game (ZPG), was developed that allows for repeated assessment of prosocial behavior and for parsing the influence of reciprocity, cost, and distress on prosocial behavior. The participants' task is to navigate a virtual character through a maze and reach a treasure in a limited amount of time. Each treasure is worth 0.50 Swiss francs (∼$ 0.50). At the same time, participants see the virtual character of an ostensible co-player from another research institute in Europe who is also trying to reach a treasure. Importantly, the two players do not share the same paths in the maze and do not compete for the same treasure. Thus, in principle, the game can be played while completely ignoring the other player. Participants are told that in each round of the game they are connected via the internet with a new co-player who is sitting in a different research institute in Europe. At the onset of each round, the participant and the ostensible co-player select one of two paths. While the players move their virtual character through the maze, red and blue gates fall on the paths that can block the participant and the co-player. Each of the two players is equipped with red and blue keys with which they can open the corresponding gates. When the co-player runs out of keys, participants can use their own keys to open the gates for them. Importantly, participants cannot delay their help to observe the progression of the game (i.e., whether they need their keys themselves) as the virtual characters become inactive before the next gate falls and thus cannot reach the treasure anymore. During each trial, participants can see how many gates are still going to fall, which and how many keys they and the co-player still possess, and how much time is left (for a screenshot of the game display, see Figure 3). When playing the game, participants wear headphones as sounds convey distress cues in the distress trials and add emphasis to events on the screen (e.g., sound when a gate is falling). Importantly, to reduce demand effects, participants are never told that the purpose of the game is to help the co-player. Instead, the instructed aim of this computer game is to reach a goal, the treasure, in a short amount of time to optimize monetary winnings.

Different trial types were introduced to probe the effect of different factors on prosocial behavior. First, to assess the influence of reciprocity on prosocial behavior, no-reciprocity and reciprocity trials were created. In the no-reciprocity trials, participants had the opportunity to help the co-player while knowing that the co-player would not have any opportunity to reciprocate as either no gates at all or no gates that the participant could not open with her own keys were still going to fall. In the reciprocity trials, participants had the opportunity to help the co-player after the co-player had helped them earlier in the trial. In these trials, participants could also see that there would not be any opportunity for the co-player to reciprocate. By designing the trials this way and by changing the co-player for each trial, we excluded the possibility of participants helping because they anticipated that they might need the co-player to reciprocate later on.

To assess the influence of helping cost on prosocial behavior, there was a low- and high-cost variant of all trial types. In the high-cost variant, participants knew that after they helped the co-player, there would be a 25% chance that they would need the donated key to reach the treasure themselves; in the low-cost variant, players knew that they could donate keys without risking needing them later themselves, the only cost in this condition being loss of time.

Finally, to investigate the effect of distress cues, when the co-player's virtual character was blocked, it either a) started to cry and sweat, as implemented by visual changes in the virtual character and by crying sounds that participants heard over headphones (distress cues) or b) gave no distress cues.

This resulted in a 2×2×2 factorial design with the three factors reciprocity (no reciprocity, reciprocity), cost (low, high) and distress (no distress, distress).

One game consisted of nine trials, one of each type, plus one trial in which no helping was necessary to reduce the affordance of the game. Trial types appeared in random order with the restriction that the first reciprocity trial could appear at the earliest as the third trial. This restriction was introduced to reduce the likelihood of an anchoring effect (helping agreed upon behavior) being introduced by experiencing a helpful co-player right away. At the beginning of the game, participants were first given written and verbal instructions and asked five questions probing their comprehension of the game. Then participants completed four practice trials to familiarize themselves with the handling of the game and to determine individual reaction time thresholds. To offset individual differences in speed and proficiency with computer games, the individual time limit for all trials of a given game was set at the average time the individual required to reach the treasure in the four practice trials plus 5 s. Furthermore, to control for the possibility that interindividual differences in helping might be due to differences in participants' allocation of attention to their own and the co-player's path, we let a star appear randomly on some trials. The star yielded 0.20 Swiss francs (∼$ 0.20) when picked up. This was expected to result in the allocation of attention to the whole display, as the star could appear anywhere. If participants collected stars but refrained from helping, attentional influences on helping behavior could most certainly be ruled out.

#### Risk perception control

As interindividual differences in the behavior in the high cost trials could be brought on by differences in risk preferences, i.e., participants differ in their perception of the risk of not reaching the treasure in the high cost trials, we assessed risk preferences. First, we asked participants on an eight-point scale how risk-seeking they are and, second, we presented them with seven lotteries where the amount that can be won (6 Swiss Francs) stays the same but the amount that can be lost varies (1–7 Swiss Francs). Participants can decide for each lottery whether they want to play it or not. The computer then randomly picks one lottery and the outcome of this lottery is paid out to the participants if they had decided to play it. The number of lotteries accepted is an index for risk preferences.

#### Engagement with the game

Participants were asked after playing the game to indicate on a five-point scale how engaged they were when playing the game. A high engagement of the participants would indicate that they were emerged in the game and diminish the probability that demand effects and strategic decision-making influenced prosocial behavior in the ZPG.

#### Dictator game

To assess the convergent validity of the ZPG, participants played the dictator game [31]. Based on our reasoning about norm-based and compassion-based prosocial behavior in the introduction, we did not expect an exceedingly high correlation between the ZPG and the dictator game, but still, as both tasks assess variants of prosocial behavior, a sufficiently high correlation to maintain that the ZPG indeed measures prosocial behavior. Participants were again told that they would be paired with another player from another research institute in Europe. In the dictator game, based on random assignment, participants are endowed with 80, 120, or 160 points that they can split between themselves and an ostensible co-player who has no points. Points are later converted to money with a conversion scheme of one point equaling six, four, or three Swiss rappen (or “Swiss penny”), respectively.

#### Memory task

To assess the divergent validity of the ZPG, we used a memory task that was later used as an outcome measure for the memory training group in the intervention experiment. Participants were presented with 34 words on the computer screen and were asked to memorize them and their sequence. Each word appeared for four seconds and words were separated with a 2-sec presentation of a crosshair. After the presentation, a wordfile opened and participants had five minutes to remember as many words as possible in the correct sequence.

#### Effectiveness measures – Experiment 2

To measure the effectiveness of the compassion-training workshop, we assessed the difference in mood and compassionate feelings reported before and after compassion training. Participants completed the Positive and Negative Affective Scale [PANAS; 47] and the Compassionate Love Scale [CLS; 48]. If effective, compassion training should lead to increases in positive mood, compassionate feelings, and possibly to a decrease in negative mood.

### Experiment 1

#### Procedure

All participants gave written informed consent after having received a description of the study. They were told that they would play interactive computer games via the internet with other participants in different research institutes across Europe in order to investigate cross-cultural differences in interpersonal behaviors. In reality, there were no co-players; the ostensible co-players' behavior was pre-programmed. Participants were seated in front of a computer and the experimenter provided oral and written instructions to the Zurich Prosocial Game (see below). Participants then answered five questions testing their comprehension of the game. The experimenter then checked the answers to ensure that participants fully understood the rules of the game. Then participants put on headphones and the experimenter ostensibly logged the participant into the game network. An abbreviated version of the instructions appeared on the computer screen and participants were asked to enter a freely chosen nickname to play the game. When ready, participants started the game and played four practice rounds after which they again had the opportunity to ask questions before playing the actual game. After finishing the Zurich Prosocial Game, the experimenter provided oral and written instructions explaining the dictator game (see below) and ostensibly logged the participant into the game network again. After playing the dictator game, participants completed a memory task (see below), filled out questionnaires (see below) and completed a lottery task to assess risk preferences (see below). Participants also completed a task in the magnetic resonance (MR) scanner and other unrelated tasks (results to be reported elsewhere). All participants were debriefed after the study was completed.

### Experiment 2

### Procedure

Participants came to the lab one to two weeks prior to the training for their pre-training measurement (pre-test) and two to five days after the training workshop for their post-training measurement (post-test). The pre- and post-training measurements were identical, except that risk-preferences were only assessed at pre-test. A detailed description of the measurement procedure with respect to the ZPG can be found in the documentation of Experiment 1. Briefly, participants first played the ZPG and the dictator game, both under the assumption that they were playing the games with other participants in research institutes across Europe. Afterwards, participants completed the memory task and a lottery task to assess risk preferences (only at pre-test). Then, in contrast to the validation sample in Experiment 1, at post-test participants filled out questionnaires that were to probe the effectiveness of the compassion training (see below). Participants also completed a task in the MR scanner and other unrelated tasks (results to be reported elsewhere). Thus, the newly developed Zurich Prosocial Game was assessed in the context of several other non-helping tasks, which further helped to reduce possible demand effects of the compassion training. Participants were asked to continue praticing after the training (see below for training details) in the days before post-test. To facilitate continuation, we offered a one-hour guided evening training session on each of these days. Participants were debriefed after the end of the study.

#### Compassion and memory training

The compassion group attended a one-day training to learn a compassion- enhancing technique developed in Buddhist contemplative traditions. This compassion meditation technique (called “Metta” in Pali) aims to foster an attitude of loving kindness, emotional positivity, benevolence, and friendliness towards oneself and others [20], [58]. An experienced meditation teacher with over ten years of teaching experience led the training workshop. The training involves sitting in an upright position and developing warm, positive feelings sequentially towards oneself, a beloved person, a neutral person, a person one has difficulties with, and all human beings by imagining each while silently repeating sentences like “May you be happy” or “May you be safe” and cultivating these positive emotional attitudes towards the visualized persons. The training day was held in silence and lasted for six hours in which mental training was sometimes done while sitting and sometimes while walking. The mental training periods were usually between 15 and 30 minutes long. There was a 45 minutes lunch break in between. During the course of the training, the target of the compassion meditation changed in the following succession: oneself, beloved person, neutral person, difficult person, all human beings. Ultimately, this should lead to an attitude of emotional positivity, benevolence, and friendliness towards oneself and others [58]. Thus, as in compassion-focused therapy [7], compassion here is trained as a skill. In contrast to a momentary induction of empathic concern through instruction to feel for a specific person in distress in a specific situation, compassion training aims at permanently changing one's motivation and attitude towards others in general.

The memory control group underwent a one-day training workshop in the method of loci, a technique used to memorize items in an ordered sequence [59], [60]. An experienced memory technique teacher with over ten years of teaching experience led the training workshop. The method of loci involves linking a series of locations (e.g., a learned route through Zurich) with a series of specific items (a list of words) by creating visual mental images that combine each item with a location. For example, in order to remember the word “egg,” one would imagine a big fried egg hanging down from the towers of the cathedral in Zurich. During recall, one recreates the images by mentally walking from one location to the next. This particular mnemonic technique was chosen for the control group as it contains most elements also needed in the compassion training workshops: People need to actively engage in inner mental processing and to create active mental images and specific associations between items. The difference is that the memory group focuses purely on improving cognitive rather than affective skills. Participants of both groups were asked to continue training in the days before post-test (1–3 days) and keep a diary of their practice. They were asked to join the daily offered one-hour evening training sessions or, if this was not feasible, to train at home.
